# E-Questionnaire on health knowledge, attitudes and practices (KAP-Health) for Brazilian students in distance learning

**DOI:** 10.1080/16549716.2022.2153441

**Published:** 2022-12-23

**Authors:** Jane Biscaia Hartmann, Amanda Tribulato Rego, Julia Vieira Khoury, Marcelo Picinin Bernuci, Mirian Ueda Yamaguchi

**Affiliations:** a Postgraduate Program in Health Promotion, UNICESUMAR, Maringá, Brazil; b Department of Medicine, UNICESUMAR, Maringá, Brazil; c Postgraduate Program in Health Promotion, UNICESUMAR, Cesumar Institute of Science, Technology and Innovation, ICETI, Maringá, Brazil

**Keywords:** Online teaching, health promotion, student health, health policy, health behaviour

## Abstract

**Background:**

The role of universities in health promotion has been strengthened by institutional policies and the Health Promoting Universities movement. Together they seek to identify behaviours that are harmful to the health of university students and implement programs or interventions to promote the health of students within the physical environment of universities. However, the COVID-19 pandemic rapidly increased the number of students learning by distance. Under these circumstances, there is an increased need to assess the knowledge, attitudes and health practices of students in distance learning.

**Objective:**

To develop a KAP (knowledge, attitude and practice) questionnaire on the health of distance learning students.

**Methods:**

Development was carried out in four stages: 1) literature review to identify existing health programs in universities; 2) formulation of questions based on WHO theoretical frameworks, National Health Promotion Policy and the literature review; 3) selection of interdisciplinary expert judges and submission of the KAP-Health questionnaire for analysis of appearance, efficiency/consistency, clarity/understanding, pertinence of content and sequence of items; and 4) content validation by applying the content validity coefficient (CVC).

**Results:**

The mean CVCs obtained were all equal to or greater than 0.96, 0.95 and 0.93 for the Knowledge, Attitudes and Practices domains, respectively; all comfortably above the cut-off score of 80% (CVCt = 0.80).

**Conclusions:**

At this stage of the study, the judges consider the content of the KAP-Health questionnaire adequate to identify issues related to the health of students in distance learning. However, it is important to note that the next step is to test the usefulness of this questionnaire. We believe that our KAP-Health instrument is both original and useful for planning institutional policies in order to implement assertive strategies to promote the health of remote-learning students in Brazil and in other parts of the world.

## Introduction

In Brazil at the end of the 1980s, poverty was understood to be the main cause of learning difficulties and low school performance for children and adolescents, which generated high rates of school-year repetition, school dropouts and negative psychological consequences for the students and their families. Consequently, this led to unfavourable repercussions for the social and economic development of the nation [1].

In the light of this, the Health Promoting School (HPS) policy was created as a strategic policy for health promotion in the school environment. It has the aim of promoting, encouraging and aiding the acquisition of personal and social skills, in order to create positive values and attitudes about health, and develop the capacity for personal decision-making and participation [2]. The HPS policy aims to make the school not only a teaching-learning centre for formal content, but also to transform it into a space with a favourable physical and emotional environment for the application of health promotion programs for students and the entire school community, including their family members [3–6].

Although the system of basic education in Brazil continues to present characteristics inherent to developing countries, such as the effects of inequality in income distribution, the results obtained in the implementation of the health promoting schools policy ensured the evolution of a more sophisticated approach to health education that is more likely to positively influence the health behaviour and lifestyle of young people [4].

The provision of places in higher education in Brazil has improved significantly in recent times, stimulated by the National Education Plan 2014–2024, which aims to increase enrolment in higher education by 50% by the year 2024 [7]. This increase in the number of students in higher education led to the initiation of the movement of Health Promoting Universities (HPU) in 2018 [8]. This movement began in Canada in the 1990s, with discussions to encourage the development of actions to promote health in universities through participatory approaches involving institutional educational policy and the academic community as a whole [9]. However, currently less than 1% of Brazilian universities are implementing the HPU policy. It is a huge challenge to do this, as the HPU must incorporate health into its culture, processes and institutional policies, aiming to effectively promote the health of the university community [8,10,11].

In addition to this, the enforced distance learning brought on by the advent of the Covid-19 pandemic, thrust upon the global population in early 2020, caused a large proportion of the world’s student population to experience social isolation, under a real threat to life [12]. The impact of Covid-19 restrictions on the mental health and well-being of university students was widely recognized [13,14]. High rates of depression, anxiety and suicidal thoughts were attributed to the lockdown experience [15,16] and represent some of the worst consequences of this difficult period. Students were physically and mentally affected by the lockdown, and the shift from physical person-to-person classrooms to virtual learning (online classes) increased the prevalence of psychological stress [17].

The period of the Covid-19 pandemic further impacted the traditional teaching model by causing the acceleration and expansion of access to education by distance learning within the Brazilian educational system [18]. Therefore, new challenges have arisen for the university as a health-promoting agent in the distance learning model. In order to understand the aspects that affect the health of students, it is necessary to carry out a preliminary survey to identify what students know, believe and how they act on issues related to their health [19].

In this respect, KAP surveys can identify gaps in knowledge, cultural beliefs, or health behaviour patterns, and identify information that is commonly known and attitudes that are commonly held. To some extent, they also can identify factors which influence health behaviour that are not known to most people, reasons for their attitudes, and how and why people practice certain behaviours. KAP surveys can also assess the processes and sources of communication that are essential to guide and define health promotion strategies in specific populations [20], and identify needs, problems and barriers in the health system, as well as the solutions to improve the quality and accessibility of services [21]. In this context, surveys with KAP questionnaires at universities can be designed to understand general aspects of the health of distance learning students, who live in the most diverse environments. From this preliminary understanding, institutional educational policies can be designed to meet the demands related to the health problems presented by distance learning students in the online environment and, thus, create more assertive strategies for health promotion. Therefore, this study aimed to develop an instrument to assess knowledge, attitudes and practices (KAP) about the health of distance learning students.

## Methods

**Figure 1.**
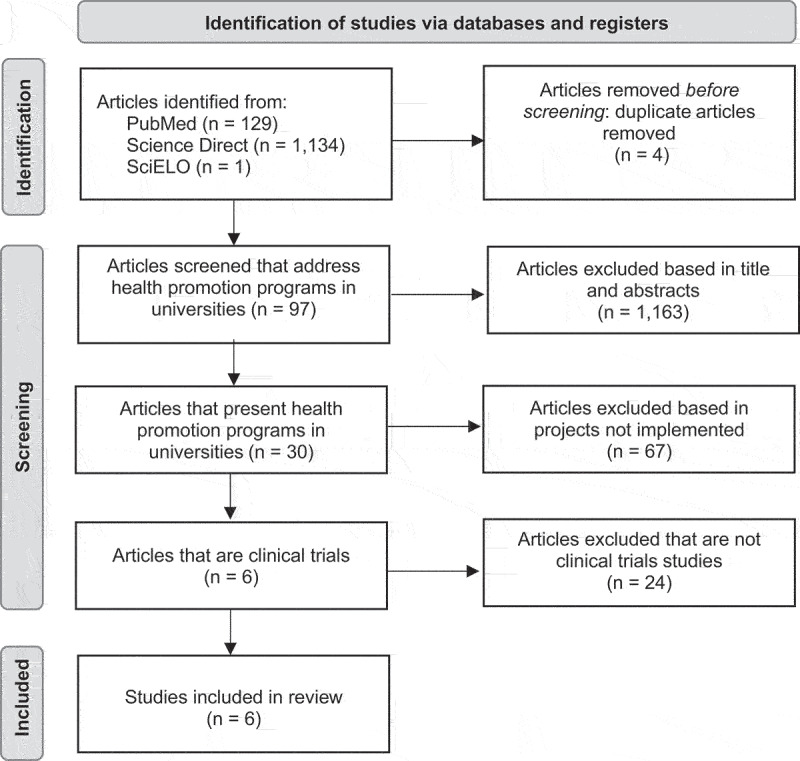
Flow diagram for literature review indicating databases and number of articles included and excluded.

This is a methodological study [22–27] for the construction and content validation of a KAP questionnaire developed in four stages. The first stage consisted of reviewing previous literature on health-promoting programs implemented in universities in the PubMed, SciELO and Science Direct databases, with the descriptors ‘students’ and ‘health promotion program.’ The inclusion criteria for this review were that they be clinical trial studies of health promotion programs carried out at universities with their students. The exclusion criterion was health promotion programs that were not implemented (Figure 1).

The second stage comprised the formulation of the questions that made up the preliminary version of the KAP-Health instrument, consisting of the domains of Knowledge, Attitudes and Practices, broadly based on the theoretical references of the World Health Organization (WHO) [28,29], Brazilian National Policy for Health Promotion [30,31] and the literature review carried out in the first stage.

The third stage consisted of the selection of expert judges with a multidisciplinary scope based on the criteria of Jasper [32], who considers that a specialist must have at least one of the following criteria: (1) skill/knowledge acquired through experience in practice or professional experience; (2) skill/specialized knowledge obtained in academic institutions with postgraduate courses; (3) special abilities gained by a particular type of study or research; (4) passing specific tests on the specialty; or (5) people who are recognized by authorities in the area and hold a high management position.

In previous literature, there is no consensus on the adequate number of judges. Lynn [33] suggests a minimum of 5 and a maximum of 10 people, chosen for their skills, qualifications and availability to participate. Considering possible unavailability or refusal to participate, the authors of this study invited 13 professionals who met at least two of Jasper’s criteria [32] and who had, on average, 15 years of experience in the area of health promotion. Table 1 shows the judges’ qualifications: sex, age, area of activity, year of graduation, postgraduate studies, professional activities, relevant positions held and other credentials.

**Table 1.** Qualification of expert judges participating in the research.

Judge sex/age	Area and year of graduation	Post-graduate studies	Professional activities	Relevant positions held and other credentials
Judge 1 F/55	Medicine (1981)	Specialization in Public Health and Hospital Administration	Lecturer	Administrative Director of Blood Bank UEM-PR-BR and Municipal Health Councilor
Judge 2 F/54	Psychology (1989)	Post-doctorate in Psychology, Doctorate in Education, Master’s in Education, Specialization in Applied Statistics	Researcher and lecturer in graduate and postgraduate courses	Director of Education in Maringa State University; Head of Dept. of Theory and Practice in Education, UEM; Co-ordinator of Graduate Program in Education, UEM-PR-BR; Evaluator of the National Institute of Educational Studies and Research, INEP/MEC; Member of the Research Group: Mental Health and Social and Environmental Contexts of Development in the Life Cycle
Judge 3 F/43	Psychology (2000)	PhD in Psychology, Master’s in Psychology (Public Health and Subjectivities)	Researcher and lecturer in graduate and postgraduate courses	Coordinator of Extension Project on Hospital Psychology. Coordinator of the Multiprofessional Team at the Regional University Hospital of UEM-Maringá, PR-BRAZIL
Judge 4 M/43	Psychology (2001)	Master’s in Psychology, Specialization in Chemical Dependence, Specialization in Special Education	Researcher and lecturer in graduate and postgraduate courses, Municipal Civil Servant in a Department of Health	Coordinator of the Psychological Care Center for university students
Judge 5 M/37	Psychology (2006)	Post Doctorate in Global Health, Doctorate in Social Psychology, Master’s in Physical Education	Researcher and lecturer in graduate and postgraduate courses	Coordinator of the Unicesumar Research Advisory Committee (CAPEC). Coordinator of the Scientific Area of the Brazilian Association of Studies in Sport and Exercise Psychology (ABEPEEx)
Judge 6 F/61	Nursing (1982)	Master’s in Nursing, Specialization in Teaching Higher Education, Municipal Management and Public Health	Municipal Civil Servant in a Department of Health	Planning Manager at the Municipal Health Department. Coordinator of Extension Project on Management and Planning in Public Health.
Judge 7 F/60	Nursing (1982)	Doctorate in Nursing, Master’s in Nursing	Researcher and lecturer in graduate and postgraduate courses	Head of Nursing Department UEM, Maringá-PR-BRAZIL. Researcher in the Project: Construction and validation of a data collection instrument for the Pediatric ICU in UEM, Maringá-PR-BRAZIL.
Judge 8 F/60	Nursing (1987)	Doctorate in Nursing, Master’s in Nursing, Specializations in Public Management, Health Auditing and Public Health.	Researcher and lecturer in graduate and postgraduate courses. Municipal Civil Servant in Department of health.	Auditor of the Municipal Department of Health, Maringá-PR-BRAZIL, Head of Basic Health Unit of the Municipality, Coordinator of the Anti-Tobacco Program of the Department of Health, Nurse Responsible for the Integrated Mental Health Center of Maringá.
Judge 9 F/58	Nutrition (1994)	Master in Health Sciences, Specialization in Montessori Method, Graduation in Pedagogy	Researcher and lecturer in graduate and postgraduate courses.	Deputy Head of the Department of Medicine at UEM-PR-BRAZIL.
Judge 10 F/63	Pharmacist (1980)	Doctorate in Public Health, Master’s in Public Health.	Researcher and lecturer in graduate and postgraduate courses.	Coordinator of the Teaching Pharmacy Program in UEM, Maringá-PR-BRAZIL, Manager of the Public Pharmacy of Brazil.
Judge 11 M/60	Dentistry (1989)	Doctorate in Public Health, Master’s in Public Health, Specializations in Service Management and Health Systems.	Director of a Health Management Consulting Company	Executive Secretary of the Brazilian Ministry of Health, State Secretary for Health in Paraná-BRAZIL and Municipal Secretary of Health in Maringá-PR-BRAZIL, President of the National Council of Municipal Health Secretaries, President of the Board of Trustees State Foundation for Health Care of the State of Paraná, Secretary of Health Surveillance of the Brazilian Ministry of Health.

The judges had an interval of 30 days to establish their considerations and suggestions on the preliminary version of the instrument. They analysed the questions of the instrument based on five criteria [34,35]: 1) Appearance: criteria attributed to the appearance and layout of the form; 2) Efficiency/Consistency: criteria attributed to the production of the desired effect and good results with regard to the reliability, accuracy and consistency of the items; 3) Clarity/Understanding: the establishment of a relationship between the transparency, perceptibility and comprehensibility of the items; 4) Content/Relevance: the relevance of the content of each item; 5) Suitable sequence of the topics and items. Additionally, a field for observations, suggestions and recommendations was provided. Each KAP-Health questionnaire question was evaluated as adequate, inadequate or in need of adjustment.

Finally, the content validation of the questionnaire was obtained by applying the content validity coefficient (CVC), proposed by Hernandez-Nieto [36], considering a minimum percentage of agreement of 80% (CVCt = 0.80). All analyses were performed using R statistical environment software (R Development Core Team), Version 3.6.2 [37].

This research was approved by the Ethics Committee in Research with Human Beings – CEP of Unicesumar, in accordance with the rules established by Resolution No. 466/12 of the National Health Council under Protocol No. 4,453,115 of 10 December 2020.

## Results

The literature review identified clinical trial studies which implemented health promotion programs carried out with students in universities in Canada [38], the United States [39], South Korea [40,41], Spain [42] and China [43]. These clinical trial studies addressed priority health issues among students, with intervention periods ranging from 10 days to 4 months. Different methodologies were used with the students to attain healthy behaviour. The study carried out by Dimeff et al. (2000) used a computer-enhanced intervention to give advice for high-risk college drinkers [39], while Pearson et al. (2013) used an interactive motivational approach with health professionals for weight loss [38]. Both studies were successful in the intervention groups.

Lee et al. (2017) combined diet and physical activity in an intensive program of just 10 days and they were successful in improving weight, BMI, muscle strength, muscle flexibility, balance, cardiorespiratory endurance and biochemical levels [41]. The Chinese study used physical activity to reduce the stress levels of students [43], and for Spanish nursing students, a positive self-control strategy improved mental health [42]. The Korean study used strategies of action planning for health promotion, which were effective for dietary practices, smoking, physical activity and self-efficacy for health management [40]. These studies addressed the health promotion themes that guided the creation of the questions in the KAP instrument developed in this study.

Of the 13 judges invited to participate in the study, one declined the invitation due to unavailability of time and a second returned the form incomplete and without signing the informed consent, making their participation unfeasible. Altogether 11 expert evaluators participated: 8 women (72.7%) and 3 men (27.3%). They had an average experience time of 31 years, with the shortest time since graduation being 15 years and the longest being 44 years. Their ages ranged from 37 to 63 years old, with an average of 54 years.

The professionals selected to be judges met at least two criteria among those proposed by Jasper [32]. In general, they had experiences related to teaching in public or private universities, or involvement in public health services in Brazil (such as the Ministry of Health), state and municipal health departments, health councils, the development of health promotion projects, and programs for the implementation and management of public health policies (Table 1).

The averages obtained in the questions and the content validity coefficient (CVC) for each domain, based on the criteria of: appearance, efficiency/consistency, clarity and understanding of the items, relevance of the content and adequate sequence of topics and items, are presented in Table 2.

**Table 2.** Content validity coefficient (CVC) of the questions following the knowledge, attitudes and practices domains.

Question	Appearance		Efficiency / Consistency		Clarity and understanding of items		Relevance of the content		Adequate sequence of topics and items	
	Mean	CVC	Mean	CVC	Mean	CVC	Mean	CVC	Mean	CVC
**KNOWLEDGE**										
1	2.91	0.97	3.00	1.00	3.00	1.00	3.00	1.00	3.00	1.00
2	2.91	0.97	2.82	0.94	2.91	0.97	2.91	0.97	2.91	0.97
3	2.91	0.97	3.00	1.00	3.00	1.00	3.00	1.00	3.00	1.00
4	2.90	0.97	3.00	1.00	3.00	1.00	3.00	1.00	3.00	1.00
5	2.82	0.94	2.91	0.97	2.82	0.94	2.91	0.97	2.91	0.97
6	2.91	0.97	3.00	1.00	2.91	0.97	2.91	0.97	2.91	0.97
7	2.82	0.94	2.91	0.97	2.82	0.94	2.91	0.97	2.91	0.97
8	2.82	0.94	2.91	0.97	2.91	0.97	2.91	0.97	2.91	0.97
9	2.82	0.94	2.82	0.94	2.82	0.94	2.82	0.94	2.82	0.94
10	2.91	0.97	3.00	1.00	3.00	1.00	3.00	1.00	3.00	1.00
11	2.91	0.97	3.00	1.00	3.00	1.00	3.00	1.00	3.00	1.00
**Mean**	**2.88**	**0.96**	**2.94**	**0.98**	**2.93**	**0.98**	**2.94**	**0.98**	**2.94**	**0.98**
**ATTITUDES**										
1	2.82	0.94	2.64	0.88	2.55	0.85	2.73	0.91	2.73	0.91
2	2.91	0.97	2.91	0.97	3.00	1.00	3.00	1.00	3.00	1.00
3	2.91	0.97	2.91	0.97	3.00	1.00	3.00	1.00	3.00	1.00
4	2.82	0.94	2.91	0.97	2.91	0.97	2.91	0.97	2.82	0.94
5	2.73	0.91	2.82	0.94	2.82	0.94	2.82	0.94	2.82	0.94
6	2.64	0.88	2.36	0.79	2.36	0.79	2.55	0.85	2.73	0.91
7	2.91	0.97	3.00	1.00	2.91	0.97	3.00	1.00	3.00	1.00
8	2.91	0.97	2.82	0.94	2.91	0.97	2.82	0.94	3.00	1.00
9	2.82	0.94	2.91	0.97	3.00	1.00	2.91	0.97	2.91	0.97
10	2.91	0.97	3.00	1.00	3.00	1.00	2.91	0.97	2.91	0.97
11	2.91	0.97	3.00	1.00	3.00	1.00	3.00	1.00	3.00	1.00
**Mean**	**2.84**	**0.95**	**2.84**	**0.95**	**2.86**	**0.95**	**2.88**	**0.96**	**2.90**	**0.97**
**PRACTICES**										
1	2.91	0.97	2.91	0.97	3.00	1.00	3.00	1.00	3.00	1.00
2	2.91	0.97	2.55	0.85	2.73	0.91	2.64	0.88	2.64	0.88
3	3.00	1.00	2.64	0.88	3.00	1.00	2.91	0.97	3.00	1.00
4	2.82	0.94	2.91	0.97	2.91	0.97	3.00	1.00	3.00	1.00
5	2.91	0.97	2.73	0.91	2.73	0.91	2.82	0.94	2.91	0.97
6	3.00	1.00	2.73	0.91	2.82	0.94	2.91	0.97	3.00	1.00
7	2.91	0.97	2.91	0.97	2.91	0.97	3.00	1.00	2.64	0.88
8	2.91	0.97	2.91	0.97	2.91	0.97	2.91	0.97	3.00	1.00
9	2.91	0.97	2.82	0.94	2.82	0.94	2.82	0.94	2.91	0.97
10	2.91	0.97	2.91	0.97	2.91	0.97	3.00	1.00	3.00	1.00
11	2.64	0.88	2.64	0.88	2.55	0.85	2.64	0.88	2.64	0.88
**Mean**	**2.89**	**0.96**	**2.79**	**0.93**	**2.84**	**0.95**	**2.88**	**0.96**	**2.89**	**0.96**

Table 2 shows that for the Knowledge domain, the CVCs were all well above the cut-off point of 0.80, at 0.96 or higher. For the Attitudes domain, both the means and the CVCs obtained in question 6 were lower in relation to the other items for all the evaluated criteria, although only the value obtained for the efficiency/consistency criterion was slightly lower than the proposed cut-off point (CVC of 0.79). However, for the Attitudes domain as a whole, the content validity was considered satisfactory in all the proposed criteria. Finally, for all items in the Practices domain, the CVCs were 0.93 or higher, which again were significantly higher than the cut-off point of 0.80.

The proposal to develop the content of the KAP-Health instrument was broadly based on the expanded concept of health from the perspective of the National Health Promotion Policy, instituted in Brazil in 2006 [30,31]. Among the priority themes of this policy are: adequate and healthy diet, bodily practices and physical activity, discouraging the use of tobacco and its derivatives, and the abusive use of alcohol and other drugs, in addition to training and continuing education. These themes correspond to studies found in previous literature, which identified health promoting interventions for the demands of students in the above-mentioned countries [38–43].

In the KAP-Health instrument, the domain related to practices included questions about searching for health information, mental health, interpersonal relationships, leisure activities, nutrition, eating habits, sleep and about the use of preventive health measures, totalling 11 questions. In the attitudes domain, that is, the inclination to think, feel and act in a particular way in relation to a certain health condition, 10 questions were created about: healthy and unhealthy habits; daily life activities and routines; and spirituality, religiosity and happiness. In turn, in the knowledge domain, 11 questions were created to identify the concepts of: health, health promotion (from nutritional, mental, physical and leisure points of view), and aspects related to health risks, such as those related to consumption disorders involving alcohol and smoking, and inadequate habits related to hours of sleep and water intake, among others, inherent in the lives of young students. The KAP-Health questionnaire is shown in Figure 2.

**Figure 2.**
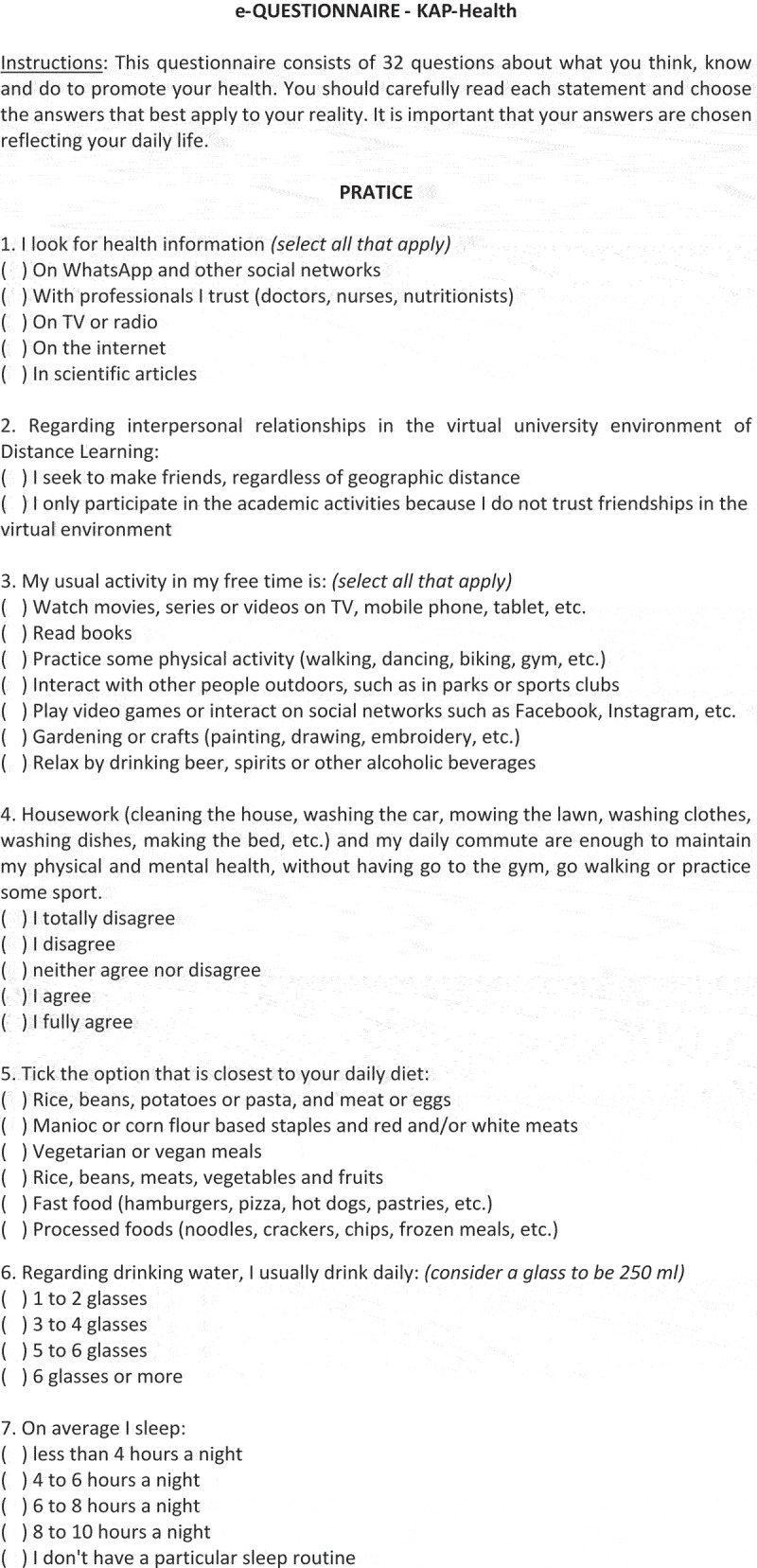
E-Questionnaire – Knowledge, attitudes and practices in health (KAP-Health).

**Figure 2.**
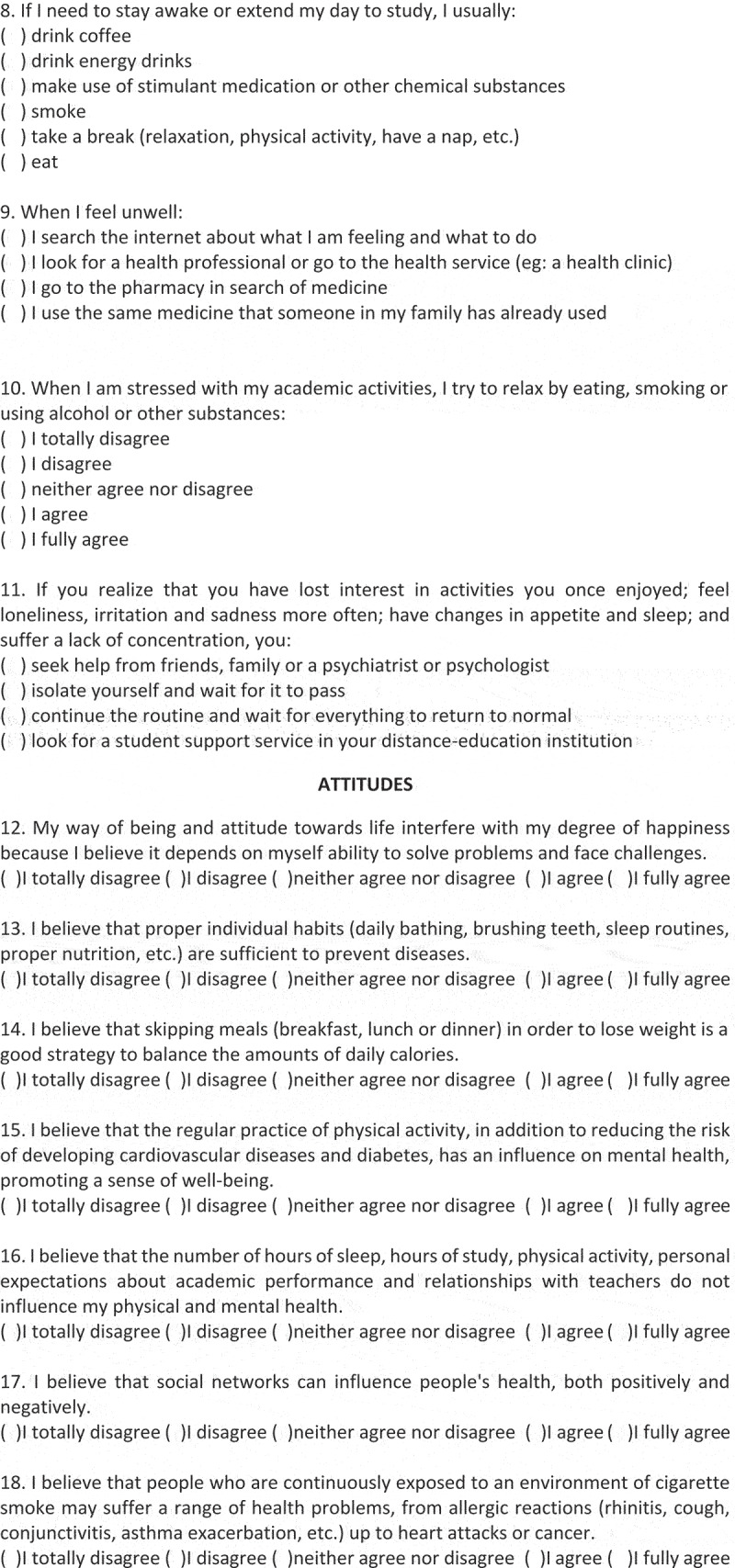
(Continued).

**Figure 2.**
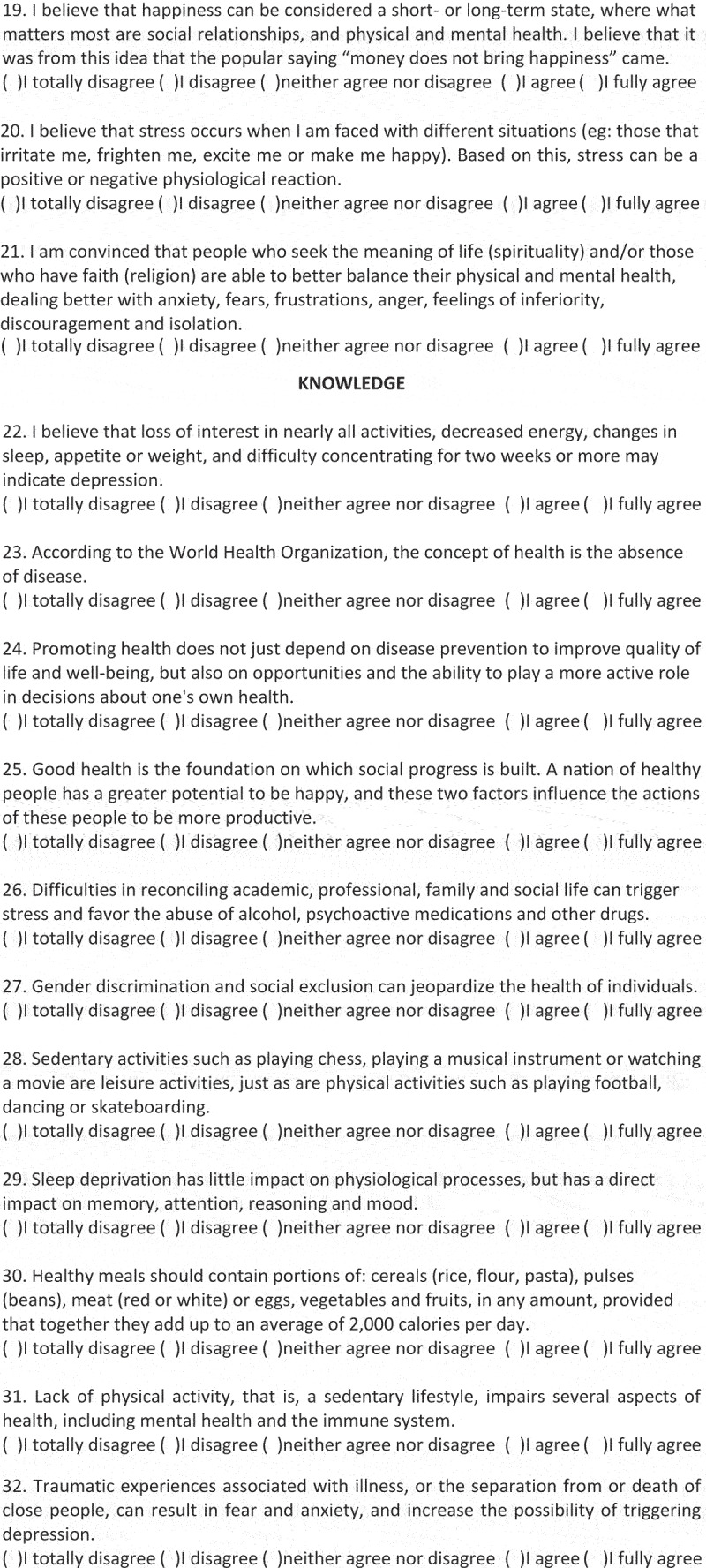
(Continued).

## Discussion

Although the number of students in distance learning was already significant before the Covid-19 pandemic in Brazil and in the world, the situation experienced in the years 2020 and 2021 dramatically increased the number of students studying in this way in comparison to face-to-face teaching [44]. In the light of this, this study sought evidence of the content validity of a KAP-Health instrument developed with the purpose of identifying the knowledge, attitudes and health practices of students in the distance learning system. The content of the instrument was evaluated by experts rigorously selected by the authors [32] and the Content Validity Coefficients (CVC) obtained indicated adequate validity of the instrument, with mean CVCs of between 0.93 and 0.98.

KAP surveys have been widely used to investigate health behaviour. The first reference to this type of research took place in the 1950s and the first study using it was published in 1968, in the field of family planning, contraceptive methods and population studies [45–47], which started to consolidate it as a widely used form of research for identifying weaknesses that necessitate strategies for health promotion. More recently, the WHO used a KAP instrument in a survey on communication and social mobilization for tuberculosis control [48], and in Brazil, a KAP study on sexually transmitted infections, AIDS and hepatitis [49] was used to investigate sexual behaviour and obtain information on health-seeking practices.

In addition, many KAP studies have been developed to understand various issues related to the knowledge and behaviour of the population during the Covid-19 pandemic [50], including studies on the mental health and general behaviours of students in the pandemic period [51]. However, to date, only our KAP instrument has been developed for distance learning students.

In the present study, it is anticipated that the KAP-Health instrument will allow the evaluation of the Knowledge, Attitudes and Practices of students in distance learning, providing information about the knowledge of students, with regard to the acquisition, retention and use of health information. Information about their attitudes make it possible to identify beliefs, feelings and preconceived ideas in relation to the subject, and information about their practices shows the way in which they demonstrate their knowledge and attitudes through their actions [52]. It is worth mentioning that a KAP study is especially useful when the knowledge and attitude are satisfactory but the practice is flawed. Often people are unable to put their health-related knowledge into practice due to economic barriers or simply because of the environment in which they are living. Therefore, health-promoting universities increasingly seek to create healthier environments, in addition to offering, for example, healthier food options on campus [9]. On the other hand, for distance learning students, the challenge seems to be greater, considering the diversity of conditions in which this population lives.

With regard to the semantic analysis of the first version of KAP-Health, the 11 judges offered a total of 49 minor qualitative suggestions relating to 9 of the questions (1, 2, 5, 7, 9, 10, 12, 14 and 17). The suggestions were analysed by the authors and 82% of them were accepted. The nine suggestions that were not considered referred to question 6, which was excluded, as we agreed with the judges, who considered it to be ambiguous in content. In addition, it was a question about digital games, which is already covered in the third question of the Practices domain.

We noticed that the judges who work with academic activities tended to make more suggestions, even when they rated a question as adequate, in comparison to the judges working in other areas. This characteristic is intrinsic to teachers who are trained to correct and improve writing [53], suggesting small observations such as substitutions of analogous terms and changes in word order, among other recommendations, that were accepted and contributed to semantically improve sentence interpretation.

In relation to the content validity of the questions, only question 6 of the Attitudes domain, which obtained CVC = 0.79, was excluded from the preliminary version. The final version of the KAP-Health instrument was structured in the following sequence: Practices domain with 11 questions, Attitudes domain with 10 questions and Knowledge domain with 11 questions. Although KAP instruments usually present questions firstly about knowledge, followed by questions about attitudes and practices, in our instrument, we first introduced the domain about Health Practices, followed by the Attitudes and then the Knowledge domains. The objective of the change in the sequence of the domains, was to mitigate the phenomenon of social desirability in the responses, which can lead to bias in self-reporting questionnaires [54]. Social desirability is a propensity on the part of people to give answers considered to be more socially acceptable and to deny personal association with opinions or behaviours considered to be socially discrediting [55].

Finally, we have identified some limitations to this study. KAP surveys are widely used in low- and middle-income countries, but there is no standardized methodology for developing this type of instrument. Thus, greater conceptual rigor in contextualizing the questions that make up the questionnaire is needed, which makes the judgments made by the judges very important. In this sense, there is a second weakness to our study: that most of the selected judges were professionals who worked only in the southern region of Brazil. Therefore, the next steps for the present study will be to carry out a pilot test with Brazilian students in distance education, and then to carry out further studies in other countries to validate the global relevance of the KAP-Health questionnaire.

## Conclusion

In this study, adequate evidence of content validity was obtained for the KAP-Health instrument, developed to investigate the knowledge, attitudes and health practices of Brazilian university students in distance learning. We believe that the results of research on the health of remote-learning students in Brazil will be useful in planning more assertive institutional policies in order to support self-care strategies and personal skills to improve the quality of life of these students. Finally, considering the recent expansion of distance learning and the originality of the KAP-Health instrument, we expect that other researchers will be able to use this instrument in surveys in institutions all around the world.

## Paper context

The acceleration and expansion of distance education in the world education system has brought new challenges to universities as health promoting agents. In order to understand the aspects that affect the health of students in distance learning, the KAP-Health questionnaire was developed to assess the knowledge, attitudes and practices relating to the health of these students, enabling the identification of the issues that affect their health and, thus, helping to strengthen the institutional educational policies of universities.
